# Comparative study between minimally invasive supraorbital craniotomy and pterional craniotomy for treating anterior circulation cerebral aneurysms in a low-resource setting

**DOI:** 10.1038/s41598-021-85115-7

**Published:** 2021-03-10

**Authors:** Ricardo Brandão Fonseca, Alyne Oliveira Correia, Raysa Siqueira Vieira, José Erivaldo Fonseca dos Santos, Heverty Rocha Alves-Neto, Anajara Ferraz da Silva Vieira, Diego Ramon Ferreira Belém, Marcos Tobias-Machado, Claudio Henrique Fernandes Vidal, Jaques Waisberg

**Affiliations:** 1 Department of Neurology and Neurosurgery, University Hospital, Universidade Federal do Vale do São Francisco, Petrolina, Pernambuco Brazil; 2 Department of Surgery, ABC Medical School, Santo André, São Paulo Brazil; 3 Department of Neurosurgery, Getúlio Vargas Hospital, Recife, Pernambuco Brazil

**Keywords:** Neurology, Cerebrovascular disorders

## Abstract

The challenges encountered in performing minimally invasive approaches, such as supraorbital minicraniotomy (SOMC), in services without adequate equipment are rarely reported in the literature. This study analyzes the viability of SOMC in the treatment of cerebral aneurysms, using exactly the same resources as pterional craniotomy (PC). The results of these two techniques are compared. 35 patients underwent SOMC, compared to 50 patients underwent CP (100 aneurysms in total), using the same microsurgical instruments. The following variables were compared: operative time, angiographic cure, length of intensive care unit stay during the post-operative period, surgical complications, length of hospital stay after surgery until hospital discharge, intraoperative aneurysm rupture, aesthetic satisfaction with the scar, and neurological status at discharge. SOMC had a significantly shorter operative time in relation to PC (213.9 ± 11.09 min and 268.6 ± 15.44 min, respectively) (p = 0.0081).With respect to the cosmetic parameters assessed by the Visual Analog Scale, the average for SOMC was 94.12 ± 1.92 points, and the average for PC was 83.57 ± 4.75 points (p = 0.036). SOMC was as effective as PC in relation to successful aneurysm clipping (p = 0.77). The SOMC technique did not show advantages over PC in any other variable. Even in a general neurosurgery service lacking a specific structure for minimally invasive surgeries, SOMC was feasible and effective for treating intracranial aneurysms, using the same set of microsurgical instruments used for PC, obtaining better results in operating time and cosmetic satisfaction.

## Introduction

Of the microsurgical approaches to cerebral aneurysms in the anterior circulation of the circle of Willis, the pterional craniotomy (PC) technique continues to be the most used and widespread in the majority of vascular neurosurgery services around the world; this has been the case since the 1970s when Yasargil and Fox systematized this type of craniotomy, associating it with the microsurgical technique for treating cerebral aneurysms^1^. The constant search for less invasive surgical access, less exposure of healthy brain tissue, better cosmetic results^2^, shorter surgical time, and greater cost-effectiveness has increasingly led to minicraniotomies, also known as keyhole craniotomies, not only for cerebral aneurysm surgeries, but for other lesions of the skull base as well^3–7^.

Supraorbital minicraniotomy (SOMC) with eyebrow incision has been little used in comparison with PC in approaches to anterior circulation aneurysms in general neurosurgery services, but it has begun to play an important role as one of the primary options for minimally invasive surgery for surgical treatment of lesions of the skull base. A considerably large number of conditions may be treated by means of this access, and this number tends to increase as the technique is enhanced and surgical instruments are adapted to the procedure^8–10^.

The literature has already gathered sufficient evidence regarding the safety and efficacy of SOMC for treating anterior circulation aneurysms using appropriate microsurgical material. The studies that predominate, however, are descriptive studies of the technique or results from case series only^3–8,10,11^. There are, in fact, few studies comparing SOMC with the standard PC technique that are able to provide evidence of the possible advantages and disadvantages of one type of craniotomy in relation to the other^12–19^.

The structural differences that exist in neurosurgery services worldwide are well known. In certain regions, such as in the Northeast of Brazil, hospitals do not have an adequate structure for minimally invasive surgery, as can be found in large centers in developed countries. The lack of a drill in good condition to perform minicraniotomy, micro-instruments adapted for minimally invasive microsurgery, neuroendoscopy, and intraoperative angiography would be a factor preventing the performance of safer procedures, such as keyhole craniotomies.

The studies found in the literature, whether describing the technique or comparing techniques, occur in favorable settings, in an entirely adapted structure, with surgeries employing advanced technology.

We did not find any studies conducted in general neurosurgery services that assessed the feasibility and safety of minimally invasive techniques, such as SOMC, in unfavorable situations, as demonstrated in this work. The objective of this article, therefore, was to ascertain whether it is possible and safe to perform minimally invasive neurosurgery in general neurosurgery services, using exactly the material that has been used for PC for decades. To the best of our knowledge, this is the first comparative study whose objectives are to prove the reproducibility of SOMC in a general neurosurgery service with limitations regarding specific instruments for minimally invasive surgery, using the same set of microsurgical instruments used for PC, and to verify whether there are advantages of using the SOMC technique in relation to the PC technique.

## Methods

This is a retrospective comparative case series study evaluating qualitative and quantitative variables between two surgical techniques, PC and SOMC. The study was conducted at the University Hospital of the Federal University of the São Francisco Valley, located in Petrolina, Pernambuco, Brazil.

### Population and sample

The study was conducted with patients who were surgically treated during the period from May 2013 to June 2019; it included a total of 85 patients, 50 from the PC group and 35 from the SOMC group, with a total of 100 operated aneurysms (40 in the SOMC group and 60 in the PC group).

### Eligibility criteria

Analysis included patients diagnosed with ruptured or unruptured cerebral aneurysm in the anterior circulation. The following were excluded: patients with cerebral aneurysm who were not treated by either of the studied surgical techniques (SOMC or PC), cerebral aneurysms in the posterior circulation, and cerebral aneurysms defined as giant (diameter > 2.5 cm).

### Data collection instruments and procedures

During the period from 2013 to 2017, PC was performed on all patients by two different surgeons; from February 2017 to June 2019, only SOMC was performed by the same surgeon. Following discharge from the hospital, patients were contacted by telephone, and outpatient consultations were carried out with physical examination, interviews, and collection of data from patients’ medical records.

The following variables were studied from patients’ medical records: sex, age, topography of the aneurysm, ruptured or unruptured aneurysm, average surgical procedure time, intraoperative aneurysm rupture, surgical wound infection, cerebrospinal fluid (CSF) leaks, intracranial hematomas or contusions that required surgery (All patients underwent tomography within 24 h after surgery.), success of clipping (cure rate), average length of stay in the intensive care unit (ICU) during the postoperative period, average length of hospital stay from the postoperative period to discharge, and neurological status at discharge as assessed by the Glasgow Outcome Scale (GOS). The GOS was divided into poor (I—death, II—vegetative state, or III—severe disability) and good (IV—moderate disability or V—mild or no disability) results. Aesthetic satisfaction with the surgical scar was assessed by the Visual Analog Scale (VAS), which ranges from zero (unsatisfactory) to 100 (satisfactory). The other parameters evaluated were the presence of temporal muscle atrophy, facial nerve injury, and frontal hypoesthesia due to supraorbital nerve injury. These data were checked by physical examination of the patient during hospitalization and/or by filling out a questionnaire in the medical records. Digital angiography and/or angiotomography were used as postoperative control to define angiographic cure.

### Surgical techniques

All of the material used for both craniotomies was exactly the same. Specific micro-instruments adapted for SOMC were not available, nor were neuroendoscopy or intraoperative angiography.

#### Supraorbital minicraniotomy with eyebrow incision

**Figure 1:**
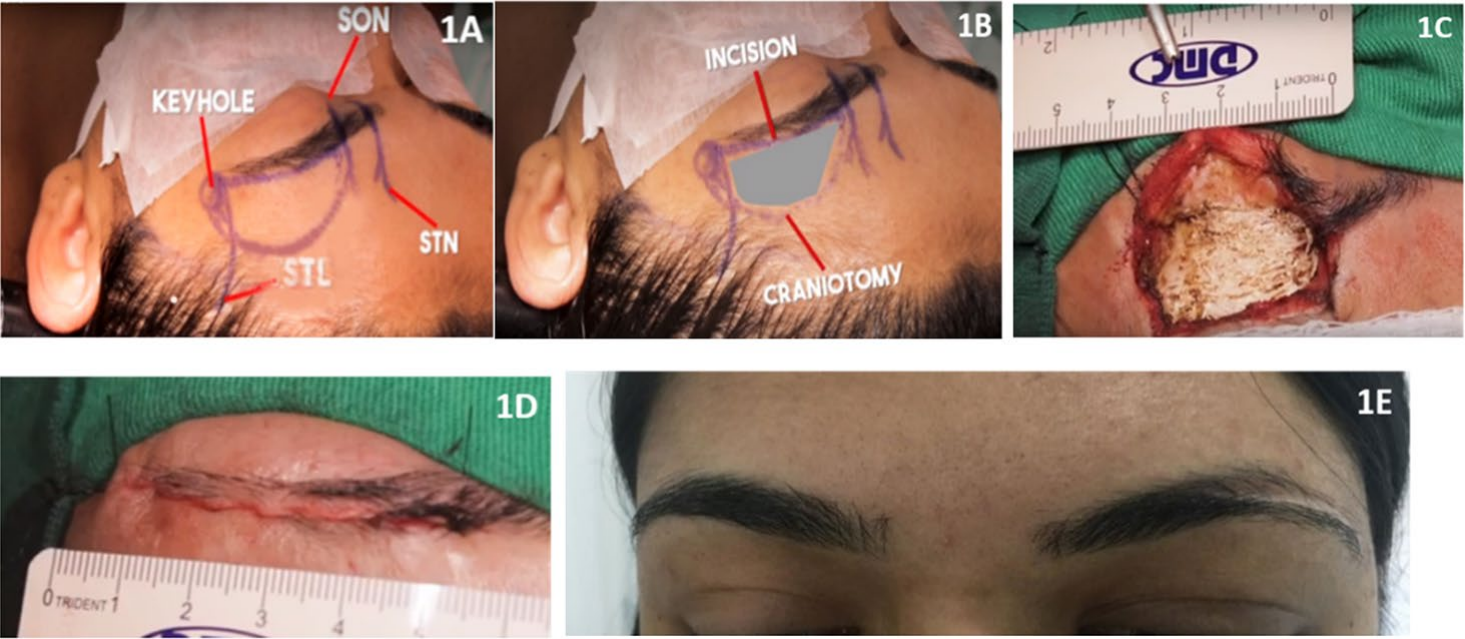
Photographs of moments of the supraorbital minicraniotomy. (**1A**, **1B**) delimitation of the incision site, craniotomy, and its adjacent structures; (**1C**) lateral displacement of the insertion of the temporal muscle, with myocutaneous flaps fixed with simple sutures, exposing the orbital ridge; (**1D**) skin closed by intradermic suture with 4.0 nylon thread; (**1E**) surgical scar after 6 months. *STL* superior temporal line, *SON*, supraorbital nerve, *STN* supratrochlear nerve (author’s archive).

The technique used was similar to the one previously described in detail by other authors^20,21^. An incision was made in the skin just above the eyebrow, laterally to the supraorbital nerve, in the midpupillary line, extending 4 to 5 cm (Fig. 1A,B). Following dissection of the subcutaneous tissue, the frontalis muscle was opened, and anterior and posterior flaps of the incision were retracted in order to enlarge the surgical field. Subsequently, slight lateral displacement of the insertion of the temporal muscle was performed for burr-hole trephination of the keyhole. The myocutaneous flaps were fixed with simple anterior and posterior sutures in the surgical field (Fig. 1C). Craniotomy was performed bordering the orbital ridge at the extension of 2 to 3 cm long and 2 to 2.5 cm high (anteroposterior). Subsequently, the reliefs of the orbital ceiling were drilled to improve microscopic visualization, and the surgeon proceeded with the arcuate opening of the dura mater with its anterior fold. Following the microsurgical procedure, the dura mater was closed by a continuous suture with 4.0 cardiovascular prolene. The bone was fixed with titanium plates and screws, and the bone gap was filled with methyl methacrylate. When there was frontal sinus penetration, we proceeded to immediate cleaning, and, finally, we also occluded it with methyl methacrylate. The muscle and the subcutaneous tissue were reconnected with absorbable threads and the skin was closed by intradermal suture with 4.0 nylon thread (Fig. 1D). The surgical scar after six months (Fig. 1E).

#### Pterional craniotomy

This study reproduced the standard technique which has previously been described by other authors^1,13–16^. The following were performed: a slightly arcuate frontotemporal incision 2 cm behind the hairline, extending anterior to the tragus until close to the midline, dissection of the planes, identification and opening of the temporal muscle and anterior folding of the myocutaneous flap, initial burr-hole trephination of the keyhole, and another posterior burr-hole trephination at the junction between the coronary suture and the superior temporal line. Subsequently, frontotemporal craniotomy and minor sphenoid wing drilling were performed, followed by the arcuate opening of the dura mater and its anterior fold. Following the intracranial procedure, the surgeon proceeded to close the dura mater with 4.0 cardiovascular prolene and dural bone lift. The free bone fragment was fixed with titanium plates and screws, and, when necessary, the trephine holes were filled with methyl methacrylate. The temporal muscle and the subcutaneous plane were reconnected with absorbable threads. The skin was closed by continuous suture with 3.0 nylon thread.

### Statistical analysis

Statistical analysis was carried out using IBM SPSS version 20 (IBM Corp., Charlotte, NC, USA) and Prism 5.0 GraphPad (Graphpade Software Inc., La Jolla, CA, USA). Averages, standard deviations (SD), and medians were obtained. Frequency tests were performed to determine percentages; Student’s “t” test was used for comparisons between craniotomy techniques for continuous qualitative variables, and the chi-squared test (χ^2^) was used to analyze categorical variables. Results were considered significant at probability values (p) < 0.05.

### Ethical aspects

This study received approval from the institution’s Research Ethics Committee under Opinion Number 2.497.762 and certificate of submission for ethical Appraisal Number 79188017.4.0000.5196. The authors certify that all participants in this study signed the informed consent form. This study was carried out in accordance with the Declaration of Helsinki, complying with all requirements for retrospective studies involving humans.

## Results

The main results are summarized in Tables 1, 2, and 3.

**Table 1:** Demographic, angiographic, surgical, and clinical characteristics of patients who underwent supraorbital minicraniotomy (SOMC) with eyebrow incision and conventional pterional craniotomy (PC).

Operative technique	SOMC	PC	*p*
Total patients (n = 85)	35	50	
Total aneurysms (n = 100)	40	60	
**Age**			
Average ± SD	51.17 ± 16.78	52.92 ± 12.95	0.6555
Median	50	55	
**Sex (%)**			
Female	22 (62.8)	38 (76)	0.1906
Male	13 (37.1)	12 (24)	
**Topography (%)**			
ACoA	19 (47.5)	17 (28.3)	
PCoA	18 (45)	19 (31.6)	
MCA	–	18 (30)	
CB	2 (5)	3 (5)	
Ophthalmic	–	3 (5)	
Choroid	1 (2.5)	–	
Ruptured aneurysm (%)	32 (80)	44 (73.3)	0.6131
Operative time (average ± SD)	213.9 ± 64.66	268.6 ± 102.4	0.0081*
**Complications (%)**			
Intraoperative aneurysm rupture	1 (2.8)	2 (4)	0.7787
Infections	–	2 (4)	0.2312
Cure rate (%)	39 (97.5)	59 (98.3)	0.7706
Time in ICU (average ± SD)	1.65 ± 1.662	4.54 ± 6.052	0.0075*
Time from admission to surgery (average ± SD)	21.72 ± 15.56	20.34 ± 12.92	0.6544
Length of hospital stay between immediate PO and hospital discharge (average ± SD)	8.12 ± 7.699	10.69 ± 14.2	0.3592

*ACoA* anterior communicating artery, *CB* carotid bifurcation, *MCA* middle cerebral artery, *ICU* intensive care unit, *PCoA* posterior communicating artery, *PO* postoperative period, *SD* standard deviation. *Significant.

Unpaired Student’s t test and chi-squared test.

**Table 2:** Postoperative status at hospital discharge in patients who underwent supraorbital minicraniotomy (SOMC) with eyebrow incision and conventional pterional craniotomy (PC).

GOS, n (%)	SOMC	PC	p
I	3 (8.8)	6 (12.2)	0.6220
II	1 (2.9)	1 (2.0)	
III	1 (2.9)	5 (10.2)	
IV	2 (5.8)	9 (18.3)	
V	27 (79.4)	28 (57.1)	
Good	29 (85.2)	37 (75.5)	0.2774ª
Poor	5 (14.7)	12 (24.4)	

*GOS* Glasgow outcome scale.

ªp value for good vs. poor GOS. Unpaired Student’s t test and chi-squared test.

**Table 3:** Results of aesthetic evaluation of patients who underwent supraorbital minicraniotomy (SOMC) and pterional craniotomy (PC).

Aesthetic (operative technique)	SOMC	PC	*p*
Total patients (n)	17	14	
VAS (average ± SD)	94.12 ± 7.952	83.57 ± 17.81	0.0360*
Temporal muscle atrophy (%)	0	9 (64.2%)	0.0071*
Facial nerve paresis (%)	6 (35.2%)	0	0.0209*

*VAS* Visual Analog Scale, *SD* standard deviation.

*Significant. Unpaired Student’s t test.

## Operative time

**Figure 2:**
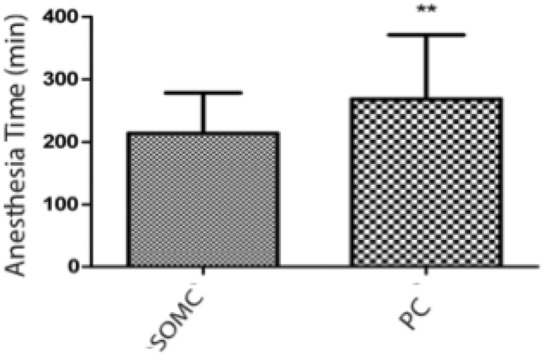
Graphs of anesthesia time (in minutes) of patients who underwent surgical treatment of cerebral aneurysms, including all aneurysms. The supraorbital minicraniotomy (SOMC) and pterional craniotomy (PC) techniques were used. The bars represent the average ± standard deviation for SOMC (n = 34) and PC (n = 44) accesses in patients who underwent aneurysm surgery (**p = 0.0081; unpaired Student’s t test).

**Figure 3:**
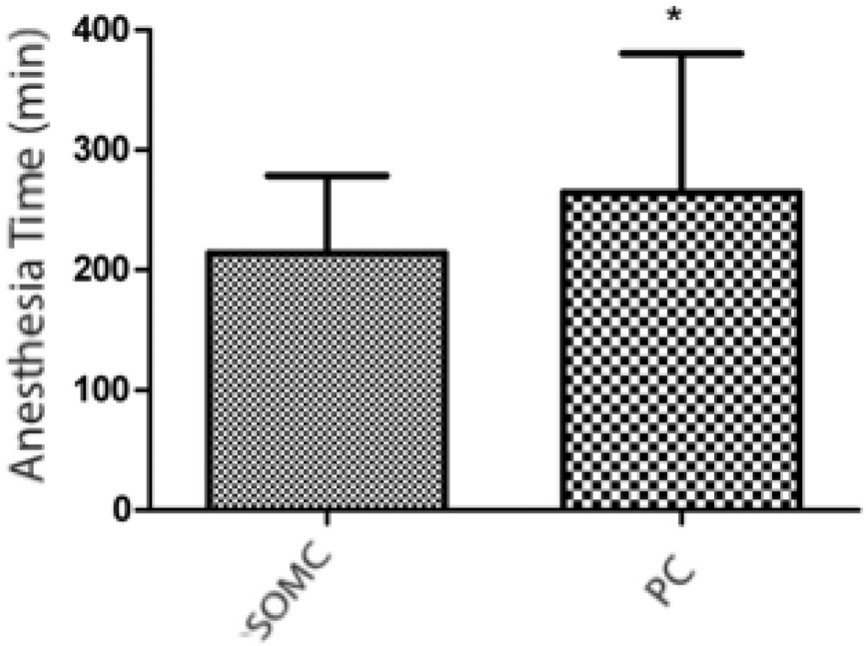
Graphs of anesthesia time (in minutes) of patients who underwent surgical treatment of cerebral aneurysms, excluding middle cerebral artery aneurysms. The supraorbital minicraniotomy (SOMC) and pterional craniotomy (PC) techniques were used. The bars represent the average ± standard deviation for SOMC (n = 34) and PC (n = 32) accesses in patients who underwent aneurysm surgery (*p = 0.0315; unpaired Student’s t test).

With the SOMC technique (n = 34) average operative time was 213.9 ± 64.66 min, and it was 268.6 ± 102.4 min (p = 0.0081) with the PC technique (n = 44) (Fig. 2). Excluding patients who underwent operation with multiple aneurysms, the SOMC approach (n = 28), with an average of 214.4 ± 62.90 min, had shorter anesthesia time than the PC technique (n = 36), with an average of 266.1 ± 110.4 min (p = 0.0308). Excluding middle cerebral artery (MCA) aneurysms, which were only operated by the PC technique, a significant difference (p = 0.0315) was also found in operative time, when comparing the two surgical techniques. For SOMC (n = 34), the average was 213.9 ± 64.66 min, and, for PC (n = 32), the average was 264.4 ± 116 min (Fig. 3).

### Surgical complications

With respect to surgical complications, intraoperative aneurysm rupture, surgical wound infections, the presence of intracranial hematomas and contusions requiring surgery, and CSF leaks were evaluated.

Of the patients who underwent SOMC, only 1 (2.8%) had intraoperative bleeding after aneurysm rupture. Of the patients who underwent PC, 2 (4%) had intraoperative aneurysm rupture (p = 0.77).

There were no surgical wound infections in the SOMC group, whereas 2 (4%) cases occurred in the PC group (p = 0.23).

There were no intracranial hematomas or contusions requiring surgery in any of the cases.

CSF leaks did not occur in any of the cases.

### Adequate clip placement: cure rate

Of the 40 aneurysms treated by SOMC, only 1 (2.5%) was not clipped, reaching a cure rate of 97.5%. With the PC technique, only 1 (1.7%) of 60 aneurysms was not successfully clipped, obtaining a cure rate of 98.3%. The difference between the two types of craniotomy was not significant (p = 0.77). With respect to the case in the SOMC group, during the intraoperative period, we realized that the aneurysm was more proximal than planned. It was a paraclinoid aneurysm, and we would have needed to remove the clinoid to approach it; with this access, we did not feel that clinoidectomy was safe, and we referred the patient for endovascular treatment.

With respect to the case in the PC group, important intraoperative hemorrhage occurred, evolving with severe cerebral edema, which made it impossible for us to approach the aneurysm and clip it; the case terminated in decompressive craniectomy, and the patient died a few days later.

### Length of total hospital stay and length of intensive care unit stay

**Figure 4:**
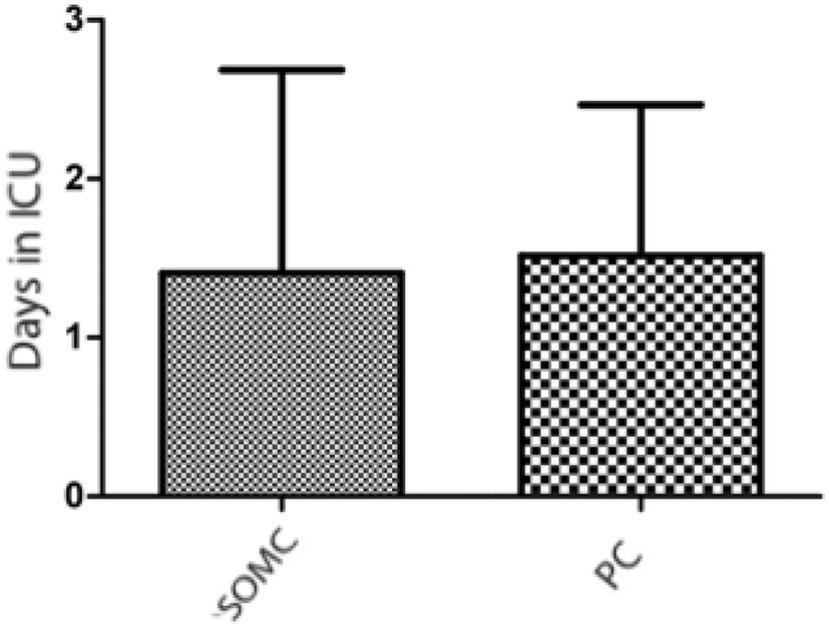
Length of intensive care unit (ICU) stay (in days) during the postoperative period in patients who underwent surgical treatment of cerebral aneurysms, excluding patients with surgical and/or clinical complications during hospitalization. The supraorbital minicraniotomy (SOMC) and pterional craniotomy (PC) techniques were used. The bar*s* represent the average ± standard deviation for SOMC (n = 27) and PC (n = 29) accesses in patients who underwent aneurysm surgery (unpaired Student’s t test; p = 0.71).

All patients were transferred to the ICU after the surgical procedure. Average length of ICU stay in the group of patients who underwent SOMC was 1.65 ± 1.662 days, while it was 4.54 ± 6.052 days (p = 0.0075) in the PC group. When analyzing only patients without surgical and/or clinical complications during hospitalization, however, no significant difference (p = 0.71) was observed in length of ICU stay (Fig. 4).

In the SOMC group, average length of hospital stay from the postoperative period to hospital discharge was 8.12 ± 7.699 days, whereas, in the PC group, the average was 10.69 ± 14.2 days (p = 0.36).

There was no significant difference (p = 0.65) between the average times from patient admission to performance of surgery (21.72 ± 15.56 days) in the SOMC group and the PC group (20.34 ± 12.92 days).

### Postoperative status at hospital discharge

In the SOMC group, 34 patients were analyzed, and 49 were analyzed in the PC group. One patient was excluded from each group due to the fact that it was not possible to occlude the lesion satisfactorily during the surgical procedure.

Patients treated by SOMC had a higher percentage (85.2%) of good GOS (IV and V) and a lower percentage (14.7%) of poor GOS (I, II, and III), compared to patients treated by PC, who had 75.5% and 24.4% good and poor GOS, respectively (p = 0.277). When only deaths (GOS I) were evaluated, 3 (8.8%) patients treated by SOMC died. Two of these deaths were not related to the neurosurgical procedure, and the other patient died due to severe vasospasm that occurred during the immediate postoperative period. In the PC group, 6 (12.2%) patients died. Two deaths were due to clinical complications; two patients died due to severe vasospasm during the immediate postoperative period, and another two died due to hemorrhagic complications. There was no significant difference (p = 0.62) between the number of deaths in the two groups (Table 2).

### Aesthetic parameters and scar complications

Aesthetic assessment was performed on 17 patients who underwent SOMC and 14 patients who underwent PC.

**Figure 5:**
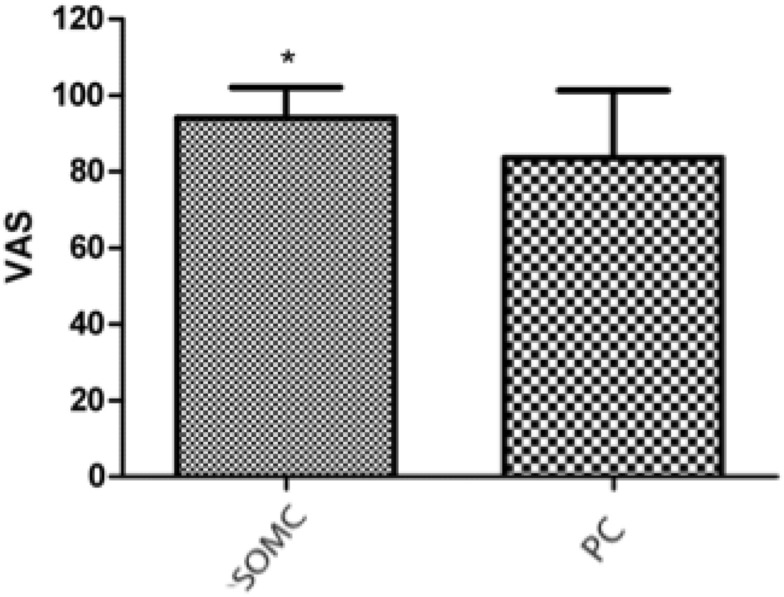
Graph of the Visual Analog Scale (VAS), in points, of patients who underwent cerebral aneurysm microsurgery, evaluated as outpatients. Supraorbital minicraniotomy (SOMC) and pterional craniotomy (PC) were used. The bars represent the average ± standard deviation of SOMC (n = 17) and PC (n = 14) accesses in patients who underwent aneurysm surgery (*p < 0.036: unpaired Student’s t test).

On the VAS, the average was 94.12 ± 7.952 for SOMC and 83.57 ± 17.81 for PC (p = 0.036) (Fig. 5).

Regarding the other evaluated parameters, 9 (64.2%) patients in the PC group had temporal muscle atrophy in the scar; in the SOMC group, there were no cases with temporal muscle atrophy (p = 0.0071). In the SOMC group, 6 (35.2%) patients had facial nerve paresis ipsilateral to the incision, while no facial paresis was observed in the PC group (p = 0.0209) (Table 3).

In the SOMC group, 11 patients reported frontal hypoesthesia, whereas only 2 patients in the PC reported it.

There were no cases of alopecia in either group.

## Discussion

Supraorbital craniotomy for treating cerebral aneurysms was initially described by Jane et al.^22^ and, subsequently, disseminated by Pernecsky and Reisch^9,20,21^.

The main objective of these less invasive approaches is not merely to make to the incision in the skin or the craniotomy smaller. The goal, in reality, is to perform an adequate access route to the skull base, while limiting trauma to the skin, the bone, the dura mater, and above all the brain^9,20^. Van Lindert et al.^21^ systematized the following two paradigms of minimally invasive cranium surgery: (1) The field of view is widened with increasing depth of the surgical target; (2) contralateral structures tend to be viewed well. The lesions closest to the opening, as a rule, require larger accesses than the lesion itself. Nonetheless, lesions located deep in the skull base can be approached by smaller craniotomies, provided that the lesions are carefully studied during the preoperative period. These principles have allowed for the enhancement of minimalist techniques for accessing the skull base.

In the present case series, a significant reduction was observed in operative time with SOMC, as shown by diverse authors^14,15,18^ and also proven by similar results in a recent meta-analysis^23^.

It is interesting to note that, although this was not a randomized study, the surgeon consecutively operated all anterior circulation aneurysms (excluding MCA aneurysms) by SOMC starting in February 2017, without selecting aneurysms by direction, size (provided that they were < 2.5 cm), or morphology. In order to obtain greater homogeneity between the two groups in the sample, the data were analyzed excluding MCA aneurysms, given that they were operated exclusively by PC; the results were, nonetheless, similar. Another relevant aspect is that the main author was the only surgeon who operated on all cases where the SOMC technique was used, which leads to less bias related to the use of this technique. The moment of surgery was also identical (p = 0.65) between both craniotomy techniques, which removes the bias of the surgery having been performed in different phases of bleeding in the two groups; similarly, there was no difference in age range between the two groups (p = 0.65).

This study used a standardized scale to measure the aesthetic variable objectively, ratifying the results found by other authors, who relate better cosmetic results for SOMC^2,12,14,18^. It is important to emphasize that there were no cases of temporal atrophy in the SOMC group, as the muscle was preserved. It could, therefore, be assumed that, in the SOMC group, there was no degree of temporomandibular dysfunction, a complaint that is quite common in craniotomies that involve opening the temporal muscle, as is the case of PC.

There was, however, a greater chance of injury to the frontal branch of the facial nerve with the SOMC technique, compared to PC (p = 0.02).

There was also a greater likelihood of supraorbital nerve injury with frontal hypoesthesia in the SOMC group. Nevertheless, this complaint was not emphasized by the patients, who only reported it when specifically asked by the examiner. None of them spontaneously complained about this sensory impairment.

In our series, there were no cases of CSF leaks or wound infections, even when there was frontal sinus penetration, given that we routinely occluded the sinus with methyl methacrylate after cleaning it. For this reason, based on our experience, we do not consider that sinus penetration would be a contraindication to supraorbital access.

In this case series, the SOMC technique was performed with the same set of instruments used in vascular microsurgery with PC (only one box of Rhoton microdissectors; only two microscissors, one curved and one straight; and two bayonet clip appliers, one standard and one mini-clip applier), which demonstrated good reproducibility of this microcraniotomy technique in services with scarce material resources.

Although the results show a significantly shorter length of ICU stay in the SOMC group, when only patients without complications during the pre- or postoperative period were analyzed, no significant difference was found between the two groups (p = 0.71), and, for this reason, the chosen craniotomy technique did not alter length of ICU stay during the postoperative period or the length of total hospital stay (p = 0.36). This result was different from the one found by Xin et al.^23^, who showed a shorter length of hospital stay in the SOMC group.

Even though some authors who use SOMC recommend using external lumbar drainage to facilitate brain relaxation and better exposure of neurovascular structures^24^, no cerebrospinal fluid drainage procedure was used in this case series. Only generous exposure of the basal cisterns was performed for cerebrospinal fluid drainage, thus avoiding external ventricular drainage and external lumbar drainage, which likely contributed to decreasing operative time and surgical morbidity, especially infectious complications, which did not occur in these patients.

This study’s results were similar to those found in a recent meta-analysis^23^ in relation to the rate of complete aneurysm occlusion and chance of intraoperative rupture, with no significant differences between the two groups. Yu et al.^18^ also found no differences in relation to the rate of intraoperative rupture between the two groups.

This study has some limitations due to the fact that it was retrospective; it was not randomized, and the sample size was limited. Furthermore, the aneurysms were not operated during the acute phase, which may have contributed to better results for the less invasive technique (SOMC) with respect to operative time. It is important to emphasize that the patients underwent surgery later, not on account of deliberate choice on the part of the surgeons, but rather due to difficulties posed by the Brazilian public healthcare system. We have a shortage of ICU beds for the postoperative period and few anesthesiologists to attend all surgical specialties; moreover, angiography exams sometimes take 7 days or more to be performed. Accordingly, it is not possible to affirm with absolute certainty how the results would have been, were they to compare patients undergoing surgery during the acute phase.

The results of this study reflect the experience of a single neurosurgery center, and they should not be extrapolated for posterior circulation cerebral aneurysms or giant aneurysms; furthermore, these results are not applicable to MCA aneurysms, given that they were operated exclusively by the PC technique.

## Conclusions

This study suggests that the SOMC technique is as safe and effective as PC for treating anterior circulation cerebral aneurysms in the non-acute phase and that aneurysms of the anterior communicating artery and the posterior communicating artery are the most appropriate for the minimally invasive technique. SOMC was shown to be feasible in a general neurosurgery service with limited resources, using the same surgical material used for traditional neurological vascular microsurgeries. There were benefits regarding surgical time and aesthetic results, and it was equivalent to PC in the other parameters evaluated. It is recommended that the minimally invasive technique be applied by surgeons who already have experience in traditional vascular microsurgery and in selected cases, as demonstrated in this study. It is, moreover, expected that these results may encourage other surgeons in services with limited resources to develop SOMC, increasingly enhancing the technique and benefiting more patients.

## Abbreviations

ACoA: Anterior communicating artery
CA: California
CB: Carotid bifurcation
CSF: Cerebrospinal fluid
cm: Centimeters
GOS: Glasgow outcome scale
ICU: Intensive care unit
MCA: Middle cerebral artery
min: Minutes
NC: North Carolina
p: Probability value
PC: Pterional craniotomy
PCoA: Posterior communicating artery
PO: Postoperative period
SD: Standard deviations
SOMC: Supraorbital minicraniotomy
SON: Supraorbital nerve
STL: Superior temporal line
STN: Supratrochlear nerve
USA: United States of America
VAS: Visual analog scale
vs: Versus
χ^2^: Chi-squared test
